# Exploring Proteases as Alternative Molecular Targets to Tackle Inflammation in Cystic Fibrosis Respiratory Infections

**DOI:** 10.3390/ijms26051871

**Published:** 2025-02-21

**Authors:** Angela Sandri, Federico Boschi

**Affiliations:** 1 Department of Diagnostics and Public Health, University of Verona, Strada Le Grazie 8-15, 37134 Verona, Italy; angela.sandri@univr.it; 2 General and Upper GI Surgery Division, Azienda Ospedaliera Universitaria Integrata Verona, Piazzale Stefani 1, 37126 Verona, Italy; 3 Department of Engineering for Innovation Medicine, University of Verona, Strada Le Grazie 8-15, 37134 Verona, Italy

**Keywords:** proteases, inflammation, cystic fibrosis, anti-protease therapy, respiratory infection

## Abstract

Cystic fibrosis (CF) is characterized by chronic respiratory infections and excessive inflammation, driven by both host- and pathogen-derived proteases. The dysregulated activity of proteolytic enzymes such as neutrophil elastase (NE), cathepsin G, and matrix metalloproteases (MMPs) degrades lung tissue, exacerbates airway remodeling, and perpetuates inflammatory cycles. Concurrently, bacterial proteases from pathogens such as *Pseudomonas aeruginosa* and *Staphylococcus aureus* contribute to immune evasion and tissue destruction, compounding disease severity. Despite advances in antimicrobial and anti-inflammatory therapies, protease-driven lung damage remains a critical challenge. This review examines the dual role of host and bacterial proteases in CF pathophysiology, highlighting emerging protease-targeted therapies aimed at mitigating lung damage and inflammation. Strategies explored include the inhibition of NE, MMPs, and bacterial proteases, with a focus on innovative therapeutic approaches such as dual-function inhibitors, biologics, and advanced drug delivery systems. By restoring the protease–antiprotease balance, these interventions offer the potential to improve clinical outcomes and quality of life for CF patients.

## 1. Introduction

Cystic fibrosis (CF) is a genetic disorder that primarily affects the lungs, resulting in chronic respiratory infections and a persistent inflammatory response that progressively damages lung tissue. Mutations in the cystic fibrosis transmembrane conductance regulator (CFTR) gene cause defective ion transport in epithelial cells, leading to thickened mucus that obstructs the airways and creates an environment favorable for bacterial colonization, particularly by *Pseudomonas aeruginosa* and *Staphylococcus aureus* [1]. These infections provoke a heightened inflammatory response, primarily driven by neutrophils, which release a range of proteolytic enzymes in an attempt to clear pathogens. The release of these proteases—especially neutrophil elastase (NE)—not only targets pathogens but also degrades host tissue, exacerbating lung injury and creating a destructive cycle of inflammation and infection [2].

The involvement of proteases in CF pathology has spurred interest in targeting these enzymes to mitigate lung damage. Host-derived proteases, including neutrophil elastase (NE), cathepsin G, and proteinase 3, play key roles in CF inflammation by degrading extracellular matrix components, impairing epithelial integrity, and amplifying pro-inflammatory signaling [3]. Elevated NE levels correlate with more severe disease outcomes, and studies suggest that chronic NE activity leads to irreversible airway remodeling and a decline in lung function [2]. Additionally, protease–antiprotease imbalance contributes to CF lung pathology, as the excessive release of neutrophil proteases overwhelms natural inhibitors like alpha-1 antitrypsin (AAT), further promoting tissue degradation [4].

Matrix metalloproteases (MMPs), a family of host proteases involved in tissue remodeling, represent another major player. Elevated MMP levels have been observed in CF airways and are associated with worse clinical outcomes, as these enzymes contribute to extracellular matrix degradation and enhance neutrophil migration, further amplifying inflammation [5].

Beyond host-derived proteases, bacterial proteases from pathogens such as *P. aeruginosa* also play a significant role in CF lung disease. *P. aeruginosa* secretes virulence factors, including elastase B (LasB) and alkaline protease (AprA), which degrade host proteins, disrupt immune signaling, and promote biofilm formation, thereby facilitating bacterial persistence and immune evasion [6]. LasB, in particular, has been shown to degrade structural proteins such as collagen and elastin, contributing to the breakdown of lung architecture and exacerbating inflammatory responses [7]. Together, host and bacterial proteases create a feedback loop of inflammation and tissue destruction, underscoring the need for targeted therapeutic approaches to disrupt this cycle and preserve lung function in CF patients.

The protease-driven cycle of inflammation and tissue destruction in CF presents a compelling case for targeted therapeutic intervention. By exploring protease inhibition, particularly of neutrophil elastase, bacterial proteases, and MMPs, researchers aim to develop treatments that reduce inflammatory damage while preserving essential immune functions. This review will explore the current landscape of protease-targeted therapies in CF, with an emphasis on their potential to mitigate lung damage and inflammation and improve clinical outcomes.

## 2. The Role of Host-Derived Proteases in CF Lung Inflammation

CF lung disease is driven by a vicious cycle of chronic infection and excessive inflammation, leading to progressive lung damage [4,5]. Among the critical mediators of this inflammatory pathology are host-derived proteases, which include serine proteases like NE, cathepsin G, and proteinase 3, as well as MMPs. These proteases, primarily released by neutrophils and other immune cells, serve essential roles in antimicrobial defense but also contribute to tissue damage and remodeling within the CF lung environment. The dysregulation of protease activity in CF exacerbates inflammation, disrupts normal tissue repair, and accelerates the decline in lung function [4,5].

### 2.1. Neutrophil Elastase (NE)

**Figure 1:**
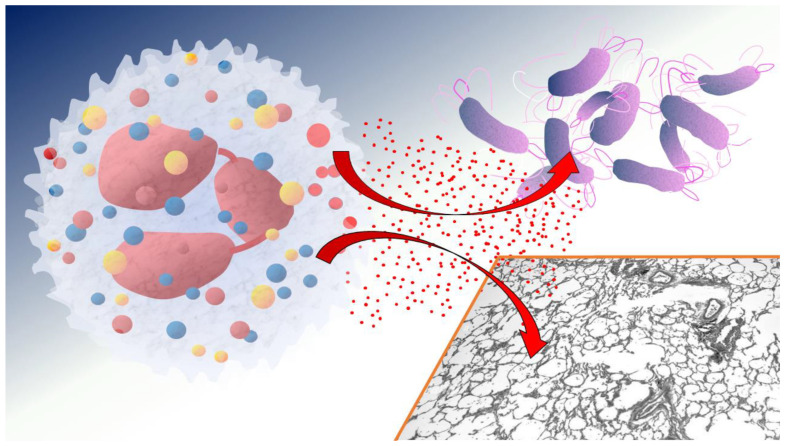
Neutrophil elastases (red points) are released from azurophilic granules of neutrophils during degranulation. It degrades bacterial cell walls (i.e., *P. aeruginosa*) but also the extracellular matrix components of the host tissue.

NE is one of the most extensively studied proteases in CF lung disease. This serine protease is stored in the azurophilic granules of neutrophils and is released into the extracellular environment during neutrophil activation or degranulation. Once released, NE has both antimicrobial functions and detrimental effects on host tissues. It contributes to pathogen clearance by degrading bacterial cell walls [8,9,10] but, unfortunately, also indiscriminately breaks down host extracellular matrix components, including elastin, collagen, and proteoglycans, leading to tissue damage and airway remodeling (Figure 1). In CF patients, elevated levels of NE have been correlated with increased disease severity, decreased lung function, and accelerated lung tissue degradation [2,11]. NE also promotes the release of cytokines such as IL-8, which further recruits neutrophils and perpetuates the inflammatory response, creating a vicious cycle of protease-driven inflammation [12,13].

### 2.2. Cathepsin G and Proteinase 3

Alongside NE, cathepsin G and proteinase 3 are two other serine proteases released by activated neutrophils that contribute to the inflammatory environment in CF. Cathepsin G is known to cleave a variety of host proteins and is implicated in enhancing neutrophil recruitment through the cleavage of CXCR2 ligands, thereby perpetuating neutrophilic inflammation [14]. Proteinase 3, another serine protease abundant in neutrophils, has similar effects, promoting cytokine release and contributing to tissue remodeling. Both cathepsin G and proteinase 3 degrade structural components of the lung tissue, and their activity is associated with impaired wound healing and lung tissue destruction [15]. The enzyme responsible for the activation of the neutrophil serine proteases is the cysteine protease dipeptidyl peptidase I (DPPI, also known as cathepsin C) [16].

### 2.3. Matrix Metalloproteases (MMPs)

Matrix metalloproteases are another group of host-derived proteases that play a prominent role in CF lung inflammation. MMPs are zinc-dependent endopeptidases that degrade various extracellular matrix (ECM) components, facilitating tissue remodeling and immune cell migration [17]. MMP-8 and MMP-9, two key MMPs involved in CF, are released by neutrophils and macrophages in response to chronic infection and inflammation [18]. MMP-9, in particular, has been shown to break down collagen, gelatin, and elastin, contributing to airway structural damage and impairing wound repair processes [19]. Elevated levels of MMP-9 are commonly found in CF patient sputum and correlate with increased disease severity and lung function decline [20].

MMPs not only degrade structural proteins but also activate other proteases and cytokines, amplifying inflammatory cascades in CF airways. MMPs can activate latent TGF-β and TNF-α, enhancing fibrotic processes and inflammatory signaling, respectively, which perpetuate tissue destruction and hinder normal repair [21,22]. Additionally, MMP-12, primarily produced by macrophages, degrades elastin and has been implicated in emphysema-like pathology in CF, further underlining the detrimental role of MMPs in CF lung disease [23,24].

### 2.4. Protease–Antiprotease Imbalance

A significant factor exacerbating CF lung inflammation is the imbalance between proteases and their endogenous inhibitors. Normally, protease activity in the lungs is tightly regulated by antiproteases such as AAT and secretory leukocyte protease inhibitor (SLPI), which inhibit NE and other serine proteases. In CF, this regulatory balance is disrupted, largely due to the overwhelming release of proteases, which outpaces the capacity of natural inhibitors [4]. Additionally, chronic infection and oxidative stress in CF airways can inactivate AAT and SLPI, further tipping the balance toward proteolytic activity [25,26]. This protease–antiprotease imbalance results in unchecked protease activity, leading to ongoing lung tissue destruction, increased mucus viscosity, and impaired ciliary function, all of which contribute to worsened lung function.

## 3. The Role of Bacterial Proteases in CF Inflammatory Pathology

In cystic fibrosis (CF), chronic lung infections by pathogens, particularly *P. aeruginosa* and *S. aureus*, play a central role in driving inflammation and tissue destruction. These bacteria secrete various virulence factors, including proteases, which disrupt host defenses, degrade extracellular matrix components, and impair immune responses, thereby perpetuating the cycle of infection and inflammation in the CF lung. The proteolytic activity of bacterial proteases not only directly damages lung tissue but also enhances the activity of host proteases, creating a synergistic effect that intensifies lung damage [27].

### 3.1. Pseudomonas aeruginosa Proteases

*P. aeruginosa* is one of the most common pathogens in CF lung infections and produces a range of proteases that contribute to its virulence. Key among these proteases are elastase B (LasB), alkaline protease (AprA), and protease IV. LasB, a metalloprotease, is particularly potent in degrading host proteins, including elastin, collagen, immunoglobulins, and cytokines, which disrupts the structural integrity of lung tissue and impairs immune responses [7]. LasB also degrades host antimicrobial molecules, thereby enhancing bacterial survival in the lung environment [28]. The activity of LasB can further augment host inflammation by promoting neutrophil recruitment and activation, which in turn leads to the release of host-derived proteases like neutrophil elastase, creating a damaging protease-rich environment [29].

AprA, another virulence factor of *P. aeruginosa*, plays a critical role in degrading immune signaling molecules, including TNF-α and IL-6, thereby impairing effective immune responses and promoting chronic infection [30]. Protease IV has been shown to degrade surfactant proteins, which are essential for lung homeostasis and innate immunity. By degrading surfactant proteins A and D, protease IV interferes with bacterial clearance and facilitates bacterial persistence in the CF lung [31].

### 3.2. Staphylococcus aureus Proteases

While *S. aureus* infections are often seen earlier in CF disease, they also contribute to inflammation and tissue damage, especially in younger patients. *S. aureus* produces several proteases, including aureolysin, which has broad-spectrum proteolytic activity against host proteins. Aureolysin can degrade complement components, impairing the host’s ability to opsonize and clear bacterial pathogens, thus allowing *S. aureus* to evade immune defenses [32]. Additionally, aureolysin can inactivate alpha-1-antitrypsin (AAT), a key inhibitor of NE, thereby further tipping the protease–antiprotease balance in favor of unchecked proteolytic activity that damages lung tissue [33].

### 3.3. Proteases of Other CF Pathogens

In addition to the well-characterized *P. aeruginosa* and *S. aureus*, several other pathogens commonly associated with cystic fibrosis (CF) lung infections also secrete proteases that contribute to the chronic inflammation and tissue destruction observed in CF. Pathogens such as *Burkholderia cepacia* complex (BCC), *Stenotrophomonas maltophilia*, and *Achromobacter* spp. produce a variety of proteolytic enzymes that may further complicate the already damaging inflammatory environment within the CF lung (Table 1).

#### 3.3.1. *Achromobacter* spp.

*Achromobacter* spp. are emerging opportunistic pathogens in CF, particularly in patients with advanced disease. These bacteria secrete a variety of proteases, such as metalloproteases, that play a significant role in host tissue destruction. *Achromobacter xylosoxidans*, one of the most common species within this genus in CF, produces proteases [40] that may degrade host extracellular matrix proteins, thus impairing tissue integrity and contributing to chronic lung inflammation, and inhibiting the activity of host immune components, further compromising the host defense and facilitating persistent infection.

#### 3.3.2. *Stenotrophomonas maltophilia*

*S. maltophilia* is another opportunistic pathogen that is frequently found in the CF lung, particularly in older patients. *S. maltophilia* produces several proteases, including serine proteases and metalloproteases, which degrade host immune factors and extracellular matrix proteins. Notably, *S. maltophilia* has been shown to produce proteases that cleave and inactivate immunoglobulins and complement factors, impairing the host’s ability to mount an effective immune response [42]. These proteases also disrupt epithelial cell integrity and promote the persistence of infection by inducing inflammation and immune cell recruitment [42,43]. *S. maltophilia* proteases may contribute to the progression of lung disease in CF by altering airway remodeling and enhancing the persistence of biofilm-associated infections.

#### 3.3.3. *Burkholderia cepacia* Complex

The *Burkholderia* genus includes several species that are significant pathogens in CF, known as *Burkholderia cepacia* complex (BCC). They produce a variety of proteolytic enzymes, including metalloproteases, which contribute to their virulence by facilitating immune evasion and tissue damage. For instance, proteases from BCC species can degrade extracellular matrix components, such as elastin and collagen, weakening the lung tissue and enhancing inflammatory responses [44].

#### 3.3.4. Other Pathogens

In addition to the aforementioned pathogens, other microorganisms produce proteases that contribute to the pathology of CF lung disease. For instance, *Haemophilus influenzae* produces serine proteases that cleave host immune factors, further compounding the inflammatory burden [41].

## 4. Mechanisms of Protease-Induced Inflammation and Tissue Damage

The interaction between host and bacterial proteases is a dynamic process that amplifies the inflammatory response in the CF lung. Host proteases such as NE and MMPs activate bacterial proteases, which in turn degrade host tissues and immune components, triggering further activation of inflammatory pathways. For example, *P. aeruginosa* proteases degrade IL-8 and IL-6, key chemokines for neutrophil recruitment, contributing to creating a paradox of ineffective inflammation that is unable to clear the infection but continues to damage lung tissue [28,45]. This synergy between host and bacterial proteases generates a vicious cycle: host proteases break down lung tissue and release cytokines, which recruit more immune cells, while bacterial proteases contribute to immune evasion and promote chronic infection. The resulting inflammation and tissue damage impede lung function, contributing to progressive pulmonary deterioration in CF patients. Importantly, this protease-driven inflammation is not only confined to structural damage but also involves mucus hypersecretion, a hallmark of CF pathology.

The overproduction of mucus in CF is primarily driven by proteases that stimulate mucin secretion from airway epithelial cells. For example, NE and other proteases can activate signaling pathways in airway epithelial cells, leading to the upregulation of mucin genes such as MUC5AC, which contributes to excessive mucus production [46]. The thickened mucus layer obstructs airways, impairs airflow, and further complicates infection clearance. Bacteria trapped in this viscous mucus layer are protected from host immune defenses and antibiotics, which, in turn, stimulate further neutrophil recruitment. This influx of immune cells increases the production of both host- and bacterial-derived proteases, worsening tissue damage and inflammation. Consequently, mucus hypersecretion and protease-driven inflammation are key contributors to the chronic, progressive nature of CF lung disease, facilitating bacterial colonization, immune dysregulation, and persistent lung damage.

## 5. Current and Emerging Protease Inhibitors

Several therapeutic strategies have been developed to inhibit protease activity in the lungs, including various classes of protease inhibitors, ranging from broad-spectrum agents to specific inhibitors targeting particular proteases (Table 2). The ongoing advancements in protease inhibition highlight a promising avenue for CF therapy, focusing on mitigating inflammation and preserving lung function.

### 5.1. Neutrophil Elastase (NE) Inhibitors

– AAT, a naturally occurring serpin, has been investigated as a therapeutic for CF due to its ability to inhibit NE. Inhaled AAT formulations have undergone clinical evaluation for CF, showing a decrease of inflammatory markers but limited reduction in sputum NE activity and poor effects on lung function [48,90]. A phase 2 trial assessed the safety and tolerability of two different doses of inhaled AAT [91]. Moreover, an anti-inflammatory role for AAT, independent of its antiprotease effect, has been suggested by Nita et al. [92].
– Sivelestat (ONO-5046) is a small-molecule NE inhibitor initially approved in Japan for acute respiratory distress syndrome. In pre-clinical studies, sivelestat appears to show benefit in acute lung injury without inhibiting the host immune defense in cases of infection. However, clinical studies on respiratory diseases do not yet provide a clear consensus [49]. In a phase III trial on mechanically ventilated acute lung injury patients with systemic inflammatory response syndrome, sivelestat improved pulmonary function, reduced mechanical ventilation duration, and shortened intensive care unit stays but had no effect on 30-day survival [50]. In the international phase II STRIVE trial, sivelestat showed no benefit in ventilator-free days or 28-day mortality [51], while a phase IV study reported a significant increase in ventilator-free days, ventilator-weaning rates, and survival rates [52].
– Alvelestat (AZD9668) is an oral NE inhibitor that has shown promise in treating lung conditions associated with elevated NE activity. In a phase 1 study for bronchiolitis obliterans syndrome (BOS) after hematopoietic cell transplantation, alvelestat was well-tolerated and showed potential in stabilizing disease progression [53]. The study, by Im et al., demonstrated improvements in FEV1 and symptom scores. Additionally, alvelestat is being explored as a treatment for alpha-1 antitrypsin deficiency-associated emphysema [54].
– Lonodelestat (POL6014) is a highly potent, selective NE inhibitor designed for inhalation. This formulation allows for direct delivery to the CF airway, achieving high local drug concentrations while minimizing systemic exposure. In early-phase clinical trials, single doses of inhaled POL6014 were safe and well-tolerated in healthy volunteers and subjects with CF, leading to high lung concentrations and NE inhibition in CF sputum, making it a promising candidate for addressing neutrophil-driven inflammation [55].
– Depelstat (EPI-hNE4) is a potent inhibitor of human NE derived from a human inter-α-trypsin inhibitor and designed to control the excess proteolytic activity in CF. Attucci et al. demonstrated that, in the sputum of CF patients, EPI-hNE4 inhibits NE resistance to hydrolysis by host proteases [56].
– BI 1323495 is a novel NE inhibitor that demonstrated high potency and selectivity in vitro, and attenuated lung damage and inflammation in a mouse model of acute lung injury [57].
– Bacterial serine protease inhibitor Ecotin was recently observed to inhibit NE enzymatic activity in CF sputa without compromising bacterial killing by neutrophils [58].

### 5.2. Matrix Metalloprotease (MMP) Inhibitors

**Figure 2:**
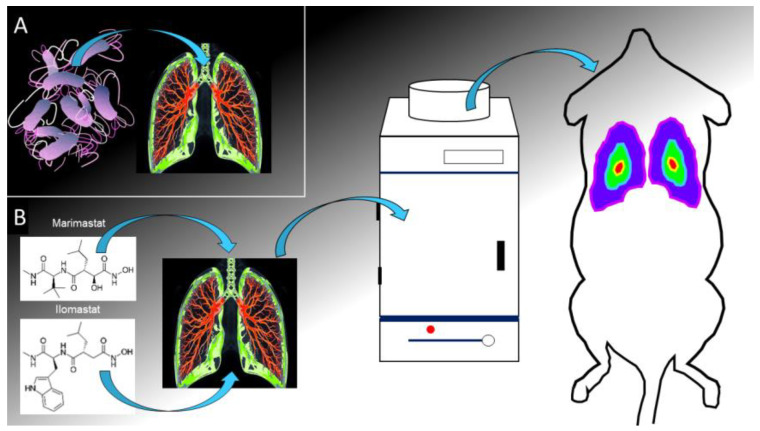
Schematic representation of the experimental protocol used to test marimastat and ilomastat metalloprotease in living small laboratory animals as done in Ref. [59]. After *P. aeruginosa* infection of lungs obtained via intratracheal instillation (**A**), marimastat or ilomastat are administered, via intratracheal instillation, in a mouse model of bioluminescent lung inflammation and the bioluminescent emission is evaluated with an optical imaging instrument (**B**). A reduction of the luminescence intensity is interpreted as an anti-MMP efficacy.

– Broad-spectrum inhibitors, such as marimastat and ilomastat, were initially developed for cancer therapy but have shown potential in reducing inflammation and tissue damage in CF models [59] (Figure 2). These inhibitors block the catalytic activity of multiple MMPs by chelating the zinc ions in their active sites. However, their clinical application has been limited by side effects, including musculoskeletal pain and off-target effects, due to the non-specific inhibition of MMPs with essential physiological roles.

– Selective inhibitors are being developed to target specific MMPs implicated in CF, for example, AZD1236, which is a selective MMP-9 and MMP-12 inhibitor. In patients with moderate-to-severe chronic obstructive pulmonary disease (COPD), two trials showed AZD1236 at 75 mg twice daily for 6 weeks was generally well-tolerated. Despite neither trial demonstrating significant short-term clinical efficacy in terms of lung function, exercise capacity, or patient-reported outcomes [60,61], possible evidence of an impact on desmosine may suggest the potential value of selective inhibitors of MMPs in the treatment of COPD in longer-term trials.

Another compound is Andecaliximab (GS-5745), an experimental medication developed by Gilead Sciences to block the function of MMP-9, aiming at reducing airway inflammation and improving lung function in CF patients. Gilead Sciences conducted a phase 2b, dose-ranging study (ClinicalTrials.gov ID NCT02759562) to evaluate the effect of GS-5745 on lung function, specifically pre-bronchodilator forced expiratory volume in 1 s (FEV1), in adult subjects with CF. However, the outcomes of this trial have not been publicly disclosed.

– Doxycycline, a tetracycline antibiotic, also acts as a non-selective MMP inhibitor by binding to the zinc ion in MMPs’ active sites. While its primary use is as an antimicrobial agent, doxycycline has been explored for its ability to reduce MMP-driven inflammation and tissue damage. In a phase 2, randomized, double-blind, placebo-controlled study by Xu et al., doxycycline significantly reduced sputum MMP-9 levels, improved lung function, and increased time to next exacerbation in CF patients during acute pulmonary exacerbations [62]. However, its potential for antimicrobial resistance limits its long-term use.

### 5.3. Bacterial Protease Inhibitors

Bacterial protease inhibitors could complement antibiotic therapy by reducing pathogenicity and improving infection outcomes in CF patients. The main approaches for bacterial protease inhibition include the following:

– Small-molecule inhibitors of bacterial proteases have shown promise in preclinical studies, reducing tissue damage and promoting bacterial clearance. For example, LasB inhibitors have been developed and tested in various models. ANT3273 demonstrated the ability to suppress IL-1β activation and reduce bacterial burden in both cellular and mouse infection models [64]. Everett et al. reported the optimization of LasB inhibitors with drug-like properties, showing their efficacy in inhibiting LasB-mediated IL-1β activation in macrophages and mouse lung infection models, as well as reducing bacterial numbers [65]. Earlier research introduced N-mercaptoacetyl-Phe-Tyr-amide as a potent LasB inhibitor, capable of blocking virulence processes, reducing biofilm growth, and enhancing the efficacy of conventional antibiotics [63].
– Aprotinin, a serine protease inhibitor, has shown promise as an antimicrobial agent against both Gram-positive and Gram-negative bacteria [66]. Aprotinin acts as a competitive inhibitor, interfering with the cleavage of antimicrobial peptides by these proteases. Ibrahim and coworkers have explored strategies to enhance the antimicrobial properties of proteins like aprotinin by creating proteolytically tailored peptides to confer the most favorable potency and specificity [67].
– Broad-spectrum inhibitors like marimastat and ilomastat, initially designed for human MMPs, have demonstrated cross-reactivity with bacterial proteases, showing a reduction of lung inflammation in CF animal models with *P. aeruginosa* lung infection [68].

### 5.4. Dual-Function and Broad-Spectrum Inhibitors

The multifactorial nature of CF lung pathology has led to the exploration of dual-function and broad-spectrum inhibitors capable of targeting multiple proteases simultaneously.

– Hybrid molecules are engineered to combine protease inhibition with antimicrobial or anti-inflammatory effects. For example, a dual-function inhibitor has been designed for the treatment of COPD, that possesses NE inhibitory activity and is also capable of attenuating inflammation by inhibiting caspase-1 [69].
– The plant-derived flavonoid epigallocatechin gallate (EGCG), a major green tea polyphenol, exhibits broad-spectrum protease inhibition alongside antioxidant and anti-inflammatory properties. EGCG has been shown to inhibit MMP-2 and MMP-9 [70,71,72], and is a potent inhibitor of NE [73]. Furthermore, EGCG suppresses reactive oxygen species (ROS) activity and inhibits apoptosis in activated neutrophils. In vivo studies by Donà et al. demonstrated that oral administration of EGCG or green tea extract enhances resolution in pulmonary inflammation [74].
– Broad-spectrum MMP inhibitors like marimastat and ilomastat, originally developed as MMP inhibitors, have demonstrated cross-reactivity with NE and bacterial proteases, highlighting their potential as multi-function agents [68,75]. By simultaneously targeting MMPs involved in tissue remodeling and NE and bacterial proteases responsible for inflammatory damage, these inhibitors may reduce both structural degradation and inflammation in CF airways [59].

## 6. Innovative Approaches in Protease Inhibition

The increasing understanding of protease-driven pathophysiology in CF has spurred the development of innovative approaches to protease inhibition. These strategies aim to overcome the limitations of traditional protease inhibitors, such as limited specificity, potential side effects, and challenges in delivering inhibitors effectively to the CF lung environment. Innovations span from engineering novel inhibitor molecules to advanced delivery systems (Table 2).

### 6.1. Small-Molecule Protease Inhibitors

Small-molecule inhibitors remain a cornerstone in protease inhibition but are increasingly being refined for greater specificity and potency. Advances in computational drug design have enabled the development of inhibitors targeting active sites and allosteric sites of proteases. For example, new-generation neutrophil elastase (NE) inhibitors are being developed with improved selectivity and reduced off-target effects, such as brensocatib (INS1007), an oral reversible inhibitor of dipeptidyl peptidase 1 (DPP-1), an enzyme responsible for the activation of neutrophil serine proteases. Initially tested in clinical trials for non-cystic fibrosis bronchiectasis (NCFBE) patients, in a phase 2 trial it prolonged the time to first exacerbation and reduced exacerbation rates by decreasing sputum NE activity, although it increased dental and skin adverse events [76]. Pharmacokinetic analyses confirmed a link between NE suppression and fewer exacerbations [77]. A phase 2b, randomized, double-blind, placebo-controlled trial (WILLOW) showed brensocatib’s broad anti-inflammatory effects by reducing multiple NSP activities [78]. A global phase 3 trial (ASPEN) is ongoing to assess its efficacy in a diverse NCFBE population [79]. In a phase 2 trial with CF patients, with and without CFTR modulators, brensocatib showed rapid absorption, dose-dependent exposure, and moderate elimination. CFTR modulators did not affect its pharmacokinetics, which were comparable to those in healthy and non-CF bronchiectasis patients. Brensocatib was well-tolerated with a safety profile consistent across groups, suggesting its potential as a treatment for CF [80].

Another DPP-1 inhibitor, BI 1291583, showed generally positive results and an acceptable safety profile in phase 1 studies in healthy volunteers [81], supporting the initiation of a phase 2 trial (Airleaf) in patients with bronchiectasis [82]. Another phase 2 study (Clairafly) is evaluating BI 1291583 for safety and efficacy in CF bronchiectasis patients [83].

### 6.2. Oxidation- and Proteolysis-Resistant Inhibitors

Secretory leukocyte protease inhibitor (SLPI) is an important respiratory tract host defense protein, which is proteolytically inactivated by excessive NE during chronic *P. aeruginosa* infection in the CF lung. NE-resistant variants of SLPI were generated by Camper et al.showing increased anti-inflammatory activity in a murine model of pulmonary *P. aeruginosa* infection [84].

Chronic neutrophilic inflammation in CF lungs also leads to excessive production of ROS, which impairs the efficacy of natural or therapeutic AAT. To address this, synthetic oxidation-resistant inhibitors are being designed, showing also improved inhibitory activity [85].

### 6.3. Biologics-Based Protease Inhibitors

A new frontier in protease inhibition is represented by antibodies. In particular, two antibodies (VH-Fc 1D1.43 and IgG1 1C10) have been developed against recombinant NE, showing specificity against NE and potent inhibition effects on its activity [86]. Instead, Kromann-Hansen et al. explored engineered nanobodies derived from camelid antibodies for targeting serine proteases [87].

MicroRNA-based therapies targeting the SERPINA1 gene, which encodes AAT, represent a novel approach to boosting endogenous AAT production in CF patients. This strategy, explored by Hunt et al., involves using antagomirs or target-site blockers to inhibit miRNAs that suppress AAT expression, potentially enhancing the antiprotease shield in CF lungs [88].

### 6.4. Advanced Drug Delivery Systems

Efficient delivery of protease inhibitors to the CF lung is critical to their success. Recent advances in inhalable drug formulations and nanotechnology have revolutionized protease inhibitor delivery. Liposomes and polymer-based nanoparticles offer a means to deliver protease inhibitors specifically to inflamed areas in the CF lung, reducing systemic toxicity and off-target effects (e.g., preserving a protease’s antibacterial action in other areas) while potentially increasing efficacy. Liposome-encapsulated aprotinin (serine protease inhibitor) has been shown by Mochalova et al. to accumulate more efficiently in the lungs, exhibit a longer residence time and, thus, has the potential for a longer therapeutic effect [89].

## 7. Challenges and Limitations

Protease-targeted therapies offer promising avenues for reducing inflammation and lung damage in CF, but several challenges limit their clinical application. Achieving high specificity remains difficult, as many inhibitors affect multiple proteases, leading to off-target effects that can impair essential functions like bacterial clearance. Additionally, the redundancy of proteases in CF means that inhibiting one may result in compensatory upregulation of others, potentially reducing overall efficacy.

Effective drug delivery to the thick, mucus-obstructed CF lungs is another barrier, as well as ensuring the stability of inhibitors in this protease-rich environment. Long-term safety is also a concern, with some therapies showing adverse effects, e.g., brensocatib caused dental and skin issues [76]. Moreover, targeting bacterial proteases poses the risk of bacterial adaptation and resistance, potentially limiting sustained efficacy.

Patient variability further complicates treatment, as differences in inflammation levels, protease activity, and infection burden necessitate more personalized approaches. Finally, regulatory and clinical development hurdles, including the need for robust long-term data and standardized protease activity measurements, add to the complexity of bringing these therapies to widespread clinical use. Overcoming these challenges will be key to translating innovative protease inhibitors into effective, long-term treatments for CF.

## 8. Future Directions

The next steps in research and development should focus on overcoming current limitations and refining therapeutic strategies. Developing highly selective inhibitors that target specific proteases, such as NE or MMPs, is essential to reduce off-target effects while preserving immune functions. In parallel, engineering oxidation- and proteolytic-resistant inhibitors that retain their activity in the environment of CF lungs could greatly enhance therapeutic efficacy.

Another promising area lies in combining protease inhibitors with other established therapies, such as antibiotics, antimicrobial peptides, or anti-inflammatory agents, to achieve synergistic effects. This approach requires careful preclinical and clinical evaluation to optimize dosing and therapeutic regimens. Personalized medicine also holds great potential, as stratifying patients based on their protease activity profiles or specific genetic and microbiological characteristics may help tailor treatments for maximum benefit.

Innovations in drug delivery systems, including nanoparticle-based formulations and liposome-encapsulated therapies, could revolutionize the targeting of protease inhibitors to inflamed areas within the lungs, reducing off-target effects and preserving other essential roles of proteases, e.g., in pathogen clearance. These advanced platforms promise enhanced localization, sustained drug release, and minimized systemic exposure. Additionally, targeting bacterial proteases, which play a central role in pathogen persistence and immune evasion, represents an underexplored but critical avenue for therapeutic intervention.

Finally, emerging technologies such as RNA-based therapeutics and engineered nanobodies provide exciting opportunities to modulate protease activity and restore protease–antiprotease balance. These multidisciplinary approaches could pave the way for transformative treatments, fundamentally altering the progression of CF lung disease.

## 9. Conclusions

Proteases are central to the pathophysiology of CF lung disease, contributing to the cycle of chronic inflammation, tissue damage, and progressive loss of lung function. The imbalance between proteases and their inhibitors, exacerbated by oxidative stress and persistent bacterial infections, underscores the urgent need for innovative therapeutic approaches.

While current protease inhibitors have shown promise, their limitations, including specificity and resistance to inactivation in the CF lung environment, necessitate further refinement. The development of next-generation inhibitors, advanced delivery mechanisms, and combination therapies offers significant potential to mitigate the inflammatory burden and preserve lung function in CF patients.

Addressing the dual contributions of host and bacterial proteases requires a comprehensive understanding of their roles in CF pathology. By integrating cutting-edge technologies and personalized approaches, future therapies could not only alleviate symptoms but also significantly improve long-term outcomes for individuals with CF.

## Figures and Tables

**Table 1:** Main proteases secreted by CF pathogens.

Microorganism	Protease	Notes	Ref.
*P. aeruginosa*	Elastase B (LasB)	Metalloprotease that degrades host proteins (e.g., elastin, collagen, immunoglobulins, cytokines, lung surfactant proteins A and D) and antimicrobial molecules.	[7,28,29,34]
	Alkaline protease (AprA)	Type 1-secreted zinc metalloprotease that degrades immune signaling molecules (e.g., TNF-α, IL-6) and fibronectin, as well as free flagellin monomers (helping immune evasion).	[30,35,36]
	Protease IV	Protease IV degrades complement proteins, immunoglobulins, fibrinogen, and lung surfactant proteins A and D.	[31]
*S. aureus*	Aureolysin	Metalloprotease with broad-spectrum proteolytic activity against host proteins (e.g., complement components, AAT).	[32,33]
	Staphopain A (ScpA)	Cysteine protease that degrades lung surfactant protein A.	[37]
BCC	Zinc metalloprotease A and B (ZmpA, ZmpB)	ZmpA and ZmpB cleave host cytokines and immunoglobulins (e.g., IFN-γ) and anti-proteases SLPI and elafin.	[38]
*S. maltophilia*	Serine proteases StmPR1, StmPR2, StmPR3	StmPR1, StmPR2, and StmPR3 degrade lung antiproteases AAT, SLPI, and elafin.	[39]
*Achromobacter spp.*	Secreted proteases	The presence of secreted proteases was recently assessed, their role in inflammation is not clear yet.	[40]
*Haemophilus influenzae*	IgA proteases	IgA proteases cleave human IgA1	[41]

**Table 2:** Quick overview of the most-often-studied protease inhibitors and their target as reported in the literature with a section dedicated to the novel approaches.

Target	Inhibitor	Note	Ref.
Neutrophil elastase (NE)	Alpha-1 antitrypsin (AAT)	Inhaled AAT formulations showed poor effects on lung function, despite a good decrease in inflammatory markers and limited reduction in sputum NE activity. Anti-inflammatory role of AAT was suggested.	[21,22,23,24,25,26,27,28,29,30,31,32,33,34,35,36,37,38,39,40,41,42,43,44,45,46,47,48]
	Sivelestat (ONO-5046)	Debated results in benefits in acute lung injury without inhibiting the host immune defense.	[49,50,51,52]
	Alvelestat (AZD9668)	Oral NE inhibitor, with elevated NE activity and well-tolerated in phase 1 study. Explored also for AAT deficiency-associated emphysema.	[53,54]
	Lonodelestat (POL6014)	Highly potent NE inhibitor, designed for inhalation, achieving high local drug concentrations while minimizing systemic exposure	[55]
	Depelstat (EPI-hNE4)	Potent inhibitor of NE, designed to control the excess proteolytic activity in CF	[56]
	BI 1323495	Novel NE inhibitor, that demonstrated high potency and selectivity in vitro, and attenuated lung damage and inflammation in a mouse model	[57]
	Ecotin	Bacterial NE inhibitor	[58]
Matrix metalloproteases (MMP)	Marimastat and ilomastat	Broad-spectrum inhibitors showing reduced inflammation and tissue damage in CF models, with clinical application limited by side effects.	[59]
	AZD1236	Selective MMP-9 and MMP-12 inhibitor, well-tolerated in patients with COPD but with low significant clinical efficacy in terms of lung function.	[60,61]
	Andecaliximab (GS-5745)	MMP-9 inhibitor, aiming at reducing airway inflammation and improving lung function in CF patients.	NCT02759562
	Doxycycline	Non-selective MMP inhibitor capable of reducing MMP-driven inflammation and tissue damage. Its antibiotic nature limits its long-term use.	[62]
Bacterial proteases	N-mercaptoacetyl-Phe-Tyr-amid	Potent LasB inhibitor, capable of blocking virulence processes, reducing biofilm growth	[63]
	ANT3273	LasB inhibitor, able to suppress IL-1β activation and reduce bacterial burden in both cellular and mouse infection models.	[64]
	Sixteen compounds	Optimization of LasB inhibitors suppressing IL-1β activation in macrophages and mouse lung infection models.	[65]
	Aprotinin	A serine protease inhibitor, has shown promise as an antimicrobial agent against Gram-positive and Gram-negative bacteria.	[66,67]
	Marimastat, ilomastat	They also demonstrated cross-reactivity with bacterial proteases, showing a reduction of lung inflammation in CF animal models	[68]
Dual function			
NE and caspase-1	Dual function molecule named “(I)”	NE inhibitory activity and is also capable of attenuating inflammation by inhibiting caspase-1 specifically designed for the treatment of COPD.	[69]
MMP-2, MMP-9 and NE.	Epigallocatechin gallate (EGCG)	EGCG has been shown to inhibit MMP-2 and MMP-9, and it is a potent inhibitor of NE. It suppresses ROS activity, inhibits apoptosis in activated neutrophils, and acts on pulmonary inflammation in in vivo studies.	[70,71,72,73,74]
NE and bacterial proteases	Marimastat and ilomastat	Cross-reactivity with NE and bacterial proteases.	[59,68,75]
Innovative Approaches			
Dipeptidyl peptidase 1 (DPP-1)	Brensocatib (INS1007)	Oral reversible inhibitor of DPP-1 (responsible for the activation of neutrophil serine proteases), it showed good results in CF patients.	[76,77,78,79,80]
DPP-1	BI 1291583	DPP-1 inhibitor, it showed good results and safety. A phase 2 trial on CF bronchiectasis is ongoing.	[81,82,83]
NE	SLPI-A16G and SLPI-S15G-A16G	Secretory leukocyte protease inhibitor (SLPI) NE-resistant variants, showed increased anti-inflammatory activity in a murine model of pulmonary *P. aeruginosa* infection.	[84]
Serine proteases and a broad group of other protease	Five alpha 1- antitrypsin constructs	Synthetic oxidation-resistant inhibitors, showing also improved inhibitory activity.	[85]
NE	VH-Fc 1D1.43 and IgG1 1C10	Two antibodies, developed against recombinant NE, showing specificity against NE and potent inhibition effects on its activity.	[86]
Serine proteases	Nanobody (Nb4)	Engineered nanobody derived from camelid antibodies explored for targeting serine proteases.	[87]
NE	MicroRNA-based therapies	Therapeutic strategies to boost levels of endogenous antiproteases such as A1AT in the CF lungs.	[88]
Serine proteases	Liposome-encapsulated aprotinin	Have been shown to accumulate efficiently in the lungs.	[89]
